# Crisis and extended realities: remote presence in the time of COVID-19

**DOI:** 10.1177/1329878X20967165

**Published:** 2021-02

**Authors:** Benjamin Matthews, Zi Siang See, Jamin Day

**Affiliations:** The University of Newcastle, Australia

**Keywords:** augmented reality, COVID-19, extended reality, future of work, mobilities, remote presence, social VR, telepresence, virtual environments, virtual reality

## Abstract

The transformative influence of the COVID-19 pandemic on remote forms of communication has been a frequent theme in popular discourse during 2020, but any lingering transformation of what we do at a distance will rely on convincing and accessible forms of remote presence and interaction. Embodied communication is difficult to simulate, and this discussion examines current and emerging extended reality (XR)–based communication tools in a range of contexts to discover what role they may play in a future where crises of mobility are likely to grow more frequent and protracted. We define XR and its current uses, then examine key terms used to conceptualise it such as ‘presence’ and ‘social presence’, before highlighting social challenges of remote presence and ethical considerations that accompany its use, particularly how the technology might (or fail to) address important social problems, support education and have relevance to the future of work.

## Introduction

Much energy has been dedicated in public discourse to the transformative influence of the COVID-19 pandemic, including consideration of whether the changes will persist. Mobilities have contracted, meaning remote forms of communication have been flung centre-stage for individuals and institutions of all kinds. For many, literacy in the use of a range of tools such as the corporate digital audio-visual meeting platform Zoom has rapidly expanded, as has the culture of their use in both work and personal spheres of endeavour. Simultaneously, the limits and advantages of these modes of communication have been explored, and it is likely future approaches to certain key areas of human interaction will be impacted by the learnings derived from this process. For instance, the costly nature of certain contexts and modes of mobilities, particularly based on air travel (including for a range of reasons such as education), may be reconsidered.

Here, the ledger is not simply weighted by considerations of economic and environmental impact, but also productivity and sociality. Any lingering transformation of what we do at a distance will rely on convincing and accessible forms of remote presence and interaction. The shortcomings of video conferencing can be identified in terms of what is missing from telepresence: the body and all it entails. Embodied interpersonal interaction is complex and our communication processes rich and difficult to simulate, and therefore challenging to achieve at a remove. This period in history has seen the product name Zoom become a verb alongside Skype, just as it has seen the emergence of phrases like ‘Zoom fatigue’ to characterise the exhaustion attenuated modes of social presence can inspire.

Some better resourced groups have made use of extended reality (XR)–based tools through this period, highlighting the limited penetration of these technologies, but also offering some insights into the efficacy and maturity of these technologies. This commentary examines the current and emerging function and sophistication of XR communication tools in a range of contexts to discover what role they may play in a future where crises of mobility such as that presently facing the human population are likely to grow more frequent and protracted. We begin by defining XR and its current uses, then attempt to examine key terms used to conceptualise it such as ‘presence’ and ‘social presence’, before highlighting key social challenges of remote presence and ethical considerations that accompany its use. Next, we provide a brief overview of significant XR service providers in the time of COVID-19, paying particular attention to how the technology might (or fail to) address important social problems, support education and have relevance to the future of work.

## What is XR?

The key areas of XR at the present time include Augmented Reality (AR), Mixed Reality (MR) and Virtual Reality (VR), and these technologies have been tested in diverse application settings such as service delivery in fitness and telehealth, clinical assessment and therapy and surgical procedures in a range of medical disciplines, education and knowledge demonstration, audio-visual entertainment and gaming, design for media production and the built environment, art and performing arts, marketing and engagement for public communications, remote meetings and collaboration in professional contexts and as part of personal social interaction.

Importantly, ‘immersion’, ‘presence’ and ‘interactivity’ are three linked criteria that allow us to distinguish between XR technologies, though the relationship between them is subject to ongoing discussion. Interactivity can be understood as ‘the extent to which users can participate in modifying the form and content of a mediated environment in real time’ (Steuer and Reeves, 1992: 84). Presence is variously defined – and we return to the complicated role of presence in understanding sociality and XR below – but most commonly understood as the feeling of ‘being there’ in a place or environment when physically located elsewhere (Skarbez et al., 2017). Immersion is a contested category, but commonly explained in terms of both presence and interactivity. Some argue immersion implies a ‘sense of presence’ (Concannon et al., 2019; Morie, 2007). Mütterlein (2018), meanwhile, argues for a complex interplay where ‘presence as well as interactivity contribute to immersion’, while ‘interactivity contributes to presence’ (p. 1407).

AR mixes live real-world visual cues with interactable digital content on mobile or wearable devices to achieve this (Billinghurst, 2011). More recent advances in AR and computing make possible a more immersive MR experience via a see-through head-mount-device (HMD), where a front facing camera provides real-time footage of the user’s surroundings (Ohta and Tamura, 2014). In defining VR, Sherman and Craig (2003) highlighted four key elements: a virtual world; immersion; sensory feedback; and interactivity. However, the longer history of attempts to define VR usually relies on the three key elements of interactivity, immersion and presence (Mütterlein, 2018). In practice, VR typically makes use of HMDs and equipment configuration with high precision tracking able to provide such things as a reliable and convincing room-scale (six degrees of freedom) user experience that includes interactive elements and multi-sensory experience of presence in a virtual world. In this way, VR aims to replicate interactable physical environments digitally.

XR-based content and applications have become increasingly distributed and employed as both professional communication tools and everyday consumer products. In scholarly research, clinical and enterprise settings, XR technologies are now moving towards a mature phase of application. They have proven useful in aiding knowledge-based demonstrations and educational experiences in a wide range of scenarios and simulations (Dey et al., 2018; Domingo and Bradley, 2018; Horst and Dorner, 2018; Marsh and Costello, 2012). XR applications have also been used in a variety of clinical and therapeutic settings and to address a range of social problems, for instance, studies have shown that VR can improve the well-being of dementia patients and assist in stress-management therapy (Guillén et al., 2018; Rose et al., 2018). VR in particular has proven useful in improving mental wellness, where it has clear relevance to those facing complex challenges such as people with a physical disability (Blum et al., 2019; Roche et al., 2019; Valtchanov et al., 2010).

XR forms the basis to a rapidly growing global media industry. SuperData, a Neilson company, revised down their estimates of global revenue from XR-related industries after the first quarter of 2020 due to the impact of COVID-19, but still project it will effectively double in under 5 years from US$6.2 billion in 2019 to US$12.2 billion in 2023 (SuperData and Quarterly Update, 2020). Of particular interest to the current discussion are platforms that allow for remote social presence, and we review exemplars and provide an overview of major commercial service providers below. As we shall see, it is noteworthy that most are in their early or beta phase of development, and at the time of writing, the majority of XR content still demands devices with high levels of computational power. In most instances, room-scale high-resolution content requires expensive computer workstations, HMDs and sensors not readily acquired by the general public. However, at the consumer level, investment in XR technologies has escalated rapidly over recent years, with the notable acquisition of VR-focussed startup Oculus by Facebook in 2014, and the subsequent release of a series of stand-alone low cost HMDs such as the gaming focussed Oculus Quest (launched September 2018), which at the time of writing retails at AU$649, or the content viewing focussed Oculus Go (launched October 2017), retailing at AU$239. Previously, a powerful computer was required to operate such HMDs, but these devices are portable and can be utilised independently and with low levels of skills acquisition.

Another significant development that will impact XR uptake is the emerging ‘AR Cloud’. The AR Cloud is, in the simplest sense, a digital copy of the world. The not for profit organisation Open AR Cloud (ndb) provides the following more detailed definition:

[I]t is a machine-readable, 1:1 scale model of the world that is continuously updated in real-time. It is a collection of billions of machine-readable datasets, point clouds, and descriptors, aligned with real-world coordinates; a living, shared, ‘soft copy’ of the world created by scanning physical features around us in which persistent augmented reality experiences reside.

This is a contested title for interlocking and major technological developments likely to have far reaching implications. It is also known by a number of other titles, including ‘Superverse’, ‘Metaverse’, ‘Mirrorworld’, ‘The Real World Web’ and ‘The Spatial Web’. Regardless of title, a contemporaneous digital copy of the world will impact XR use, and permit the delivery of myriad services via AR in particular, with indoor and outdoor navigation clear use case examples in the settings of museums, gaming, real estate and autonomous vehicles. A rash of startups including Sturfee, Scape Technologies, Visualix, YOUAR, Ubiquity6 and Niantic offer AR Cloud-based platforms for specialised navigation and marketing services. Google already offers a ‘live view’ of walking directions on Google Maps applications, which presents AR information and wayfinding anywhere good ‘Streetview’ coverage is available (Use Live View on Google Maps, nd).

However, any location-specific content delivery, gaming, maintenance and education augmentation is possible, and when coupled with the growing penetration of networked smart devices and sensors – often referred to as the Internet of Things (IoT) – the fidelity and capacity for spatial delivery of content are likely to underpin a major leap forward in XR technology. While there are clear applications for these advances in enterprise settings such as the operation of supply chains, from automated warehouses to autonomous delivery trucks, it will reach down to the consumer level. For example, a startup called Ubiquity6 launched a product in January 2020 called ‘display.land’ that permits the user to capture their surroundings using a mobile device in 3D and use that as a space for real time, shared AR and VR experiences such as small games or 3D scenes that mix the digital and real (Matney, 2020).

The achievement of such a technological leap is the subject of fierce competition, both in commercial and ideological terms. Open AR Cloud (nda) was established by a collective of smaller players to mediate this competition, and support the development of ‘open and interoperable AR Cloud technology, data and standards to connect the physical and digital worlds for the benefit of all’. However, the protocols to support complex and powerful software infrastructure are currently being defined by a number of major players, and development frameworks such as Google’s ARcore and Apple’s ARkit already exist and form the basis to a growing ecosystem of applications.

## XR and ‘presence’

Facebook has clearly positioned itself in the market to amplify penetration for their devices as gaming and content platforms, but XR also forms a part of their future plans for achieving a stronger sense of immersion during social interaction, particularly in what is commonly known as ‘social VR’. Social VR is a shared virtual environment (VE), for instance the ‘Facebook Spaces’ VR application, which is already available and permits users to interact with up to three Facebook friends (Facebook Help Center, nd). However, in each of the contexts discussed, the matter of presence – and particularly social presence – is a complicated one. Indeed, social isolation is broadly understood to be a major problem for VR and the fact that many VR applications are single user based a key reason for the slow overall market adoption rate of VR (De Simone et al., 2019; Gunkel et al., 2019; Li et al., 2019).

The presence of the body is limited by the available technology, and virtual representations of users are usually achieved via a computer-generated avatar, and Facebook’s app is currently no exception. Avatars typically offer a limited, non-lifelike version of the self or other, and despite the relative maturity of the technology arguably remains in its inchoate phase. Major research programmes from industry, such as Facebook’s own Reality Labs, and scholarly settings such as the European Commission–funded VRTogether project, are dedicated to addressing this issue by developing photorealistic representations of participants for XR use cases including VR conferencing and other shared social experiences (De Simone et al., 2019; Gunkel et al., 2019; Li et al., 2019; Rubin, 2019; VR Together, nd). Facebook, for instance, are developing ‘Codec Avatars’: photorealistic computer-generated avatars based on high-resolution image gathering and machine learning (Rubin, 2019).

Research into the use of such technologies for the purposes of collaboration has unfolded over a period of three decades, and the means by which a sense of presence can be achieved has developed far beyond ‘crude proxies’ and into more nuanced understandings of how people worked together in XR (Ens et al., 2019). In part due to investment from industry, Facebook and Apple in particular, scholars in the field are now able to shift their attention from developing the needed hardware to its application. It is likely this research will lead on to rapid improvements in immersive XR technology over the next few years.

Part of this story is a maturing capacity to conceptualise presence. A growing body of research has investigated presence and discovered the need to deepen our understanding by fine tuning the ways we approach the study of how convincing VEs are. Slater (2009) argued that realistic behavioural responses from users to situations and events portrayed in immersive VR systems can be better understood by extending on the concept of presence as ‘being there’, here known as place illusion (PI), with a second component: plausibility illusion (Psi). The last ‘refers to the illusion that the scenario being depicted is actually occurring’ (Slater, 2009: 3549). When both PI and Psi occur, the participant is more likely to respond in a realistic manner. In the virtual environment, we see a paradoxical situation unfold in which both dimensions of plausibility rely on the participant being certain they are not really there, and the scenario is not actually unfolding.

In other words, producing a plausible sense of presence calls not simply for such things as photorealistic representation but for complex subjective qualities. Slater (2009) attempts to address this through Psi, which relies on participant exposure to events that relate directly to them, and how credible the depiction of a scenario is by comparison with their expectations. While the dimension of PI is well studied, Psi is not and this is likely due to both the fact that concept is relatively new and also the complexity of its experiential quality. In a recent interview, Australian scholar Richard Skarbez explained this as follows:

[I]nvestigating plausibility illusion is just hard. PI seems to derive primarily from system characteristics: display field of view, system latency, and the like. These are quantifiable things. On the other hand, Psi derives from characteristics of the experience, such as the behaviour of objects in the environment, user’s expectations about the experience, and so on. To choose virtual fire-fighter training as an example, having a display with a wider field of view might increase a user’s feeling of PI, while having the simulated fire behave appropriately might increase their feeling of Psi. If one experiences both PI and Psi, then one is more likely to respond to virtual stimuli as if they are real. (Anjum, 2020)

In other words, to measure and understand appropriate behaviour of a fire in context from the viewpoint of a professional fire fighter is a far more complicated matter than determining the appropriate colour and shading of computer-generated flames. Now take this level of complexity, and scale it up to the setting of simulated human interaction, and we have some notion of the challenge that confronts designers working to stimulate PI and Psi for participants in immersive social telepresence XR environments.

## Social challenges of remote presence

Another aspect related to Psi is the notion of how fluid or ‘translucent’ an XR experience might be in terms of social relating (Erickson et al., 1999). As innately social beings, we constantly and subconsciously rely on myriad social cues to moderate and enhance our day-to-day interactions with others. Body language and facial expression, slight shifts in posture, variations of vocal pace, tone and loudness, use of gestures, modifying our physical distance and initiating or withdrawing from conversations are all examples of the subtle cues and tools we rely on automatically during social inputs and outputs (Clark, 1996).

Traditional video conferencing approaches such as Zoom impose substantial limits that impact the fluidity of these social experiences. As posited by Sander and Bauman (2020), and experienced firsthand by many during the recent pandemic, video conferencing can introduce a type of friction that reduces many of the verbal and non-verbal cues we rely on for fluid, natural social interactions. For example, audio lag or poor microphone hardware might force a user to speak at an uncomfortably loud volume; subtle shifts in voice tone can get lost in the digital noise; and lack of direct eye contact, hidden body language or technical hiccups such as lag can hinder the natural flow of conversation (Venter, 2019). Individually, these are typically minor annoyances that can be easily surmounted. Combined, and over extended periods, the cumulative effect of increased cognitive load can result in feelings of exhaustion and ‘Zoom fatigue’.

Relative to in-person communication, XR approaches will need to address many of these same problems to increase Psi in terms of social presence. Nonetheless, immersive, virtual environments allow users to moderate their social interactions in more fine-tuned and subtle ways and have potential to enhance social fluidity by increasing the clarity or granularity of interactions. Being able to turn towards, or move away, from another’s digital avatar; programmatically vary the loudness of others based on their proximity; or incorporate hand-like gestures, are examples of mechanisms that could facilitate a greater sense of Psi in terms of social presence (Teo et al., 2019)

## XR and inclusive design

The ethical dimension of the expanded use of these technologies must also be mentioned, and the topic is broad. There are clear dangers, for instance, in the development of photorealistic impressions of humans to become avatars, such as criminality through identity theft and negative psychophysical impacts created by self-perception and body image, such as weight perception (Thaler et al., 2018). Facebook Reality Labs recruited expert leading scholar in the field Yaser Sheikh from Carnegie Melon to assist, and he brought with him a capture technology known as the ‘Panoptic Studio’ (Joo et al., 2015). At Facebook, the updated version of this technology is known as the ‘Sociopticon’, and is made up of 180 high-resolution, high-frame-rate cameras that permit a process of machine learning to build a model of how our clothing and bodies move (Rubin, 2019). The irony of these terms for a mode of surveillance and mapping that leads to a one-to-one, high-fidelity representation of your real self will not be lost on students of history, Bentham, Baudrillard, or for that matter, Borges. If identity theft is already a problem, those problems will be amplified and particularly in their capacity to create trauma for individuals exposed to strongly immersive XR.

These dangers notwithstanding, here we focus on approaches to design going forward in order to foster a particular methodological perspective, which is centrally important during the early phases of development of scalable technologies such as those employed in XR. Cost, format and complexity of the needed hardware invite the question: how can we ensure these technologies are designed to include (and consider the needs of) the greatest number of people who might use them, while doing the least harm? And how can we use this opportunity to help people with specific needs like those with disabilities, Autism spectrum disorder, medical conditions such as obesity or dementia, diseases like Alzheimer’s, and more generally, marginalised groups in society?

Achieving the combination of PI and Psi for diverse participants throws up myriad challenges for an ethical approach to XR design, and this prospect indicates a role for inclusive design that must be human centred, rather than located in the technology. A definition for the formal concept of inclusive design can assist here. According to Kat Holmes, the term has been in use for several decades, but gained impetus when Jutta Treviranus led a team at the Ontario College of Art and Design to found the Inclusive Design Research Centre in 1993 (Holmes, 2018a). For Treviranus, the goal of an inclusive design programme of education is to produce ‘designers who have experienced barriers. What we want to produce is not a uniform set of individuals with specific competencies, but a group of individuals that can work as a team, that each can contribute a diverse perspective’ (Holmes, 2018b). Similarly, expert in inclusive design for the built environment Susan Goltsman argued that inclusive design should not be defined in terms of the attempt to create ‘one thing for all people’, but instead a ‘diversity of ways to participate so that everyone has a sense of belonging’ (cited in Holmes, 2018b).

This ethical stance need not be framed as beyond pragmatic considerations such as success in the market given the recent turn by business towards the social dimensions of enterprise. For instance, a 2019 report led by the Australian (Sydney based) not-for-profit Centre for Inclusive Design entitled *The Benefit of Designing for Everyone* was sponsored by Adobe and Microsoft, and completed in partnership with PwC Australia. It examined principles of inclusive design and highlighted that people having difficulty accessing and/or using products and services are frequently excluded from consideration in design processes with the effect of limiting the design output’s efficacy, versatility, lifespan and commercial potential (Centre for Inclusive Design, 2019).

Inclusive design approaches, they argue, that consider diverse users to drive innovation and better experiences for all participants increase the commercial potential of a product or service. Here, inclusive design is defined as

a human centred or user centred design methodology that provides a framework to understand the needs, wants, and limitations of end users. It is a methodology that encourages and employs the principles highlighted above, to enhance the reach that companies and designers have on their respective markets. (Centre for Inclusive Design, 2019)

From a human-centred perspective, therefore, we might frame inclusive design as an ethical approach that considers the various contexts of XR design as presenting separate challenges that deliver discrete solutions while demanding the consistent need to consider diverse perspectives.

## Commercial social XR in the time of COVID-19

The emerging ecology of commercial social XR technologies represents an expanding socio-technical context of communication where complex issues such as how design choices and industry practices impact sociality must be tackled. Recent research has begun to investigate, and hopes to model such intricate matters as the impacts of aesthetic presentation of virtual place and the affordances of avatars and avatar systems as mediators of embodiment, where social mechanics and the fostering of social norms that promote pro-social behaviour in social XR settings over time are key considerations (Kolesnichenko et al., 2019; McVeigh-Schultz et al., 2019).

Various established teleconference tools have been used extensively as a mode of communication in personal and professional contexts during the COVID-19 pandemic, but common platforms include Zoom, Skype, Google Meet and in the setting of education, Blackboard Collaborate. The presence-based shortcomings of these platforms have prompted existing companies with expertise in XR to engage in accelerated development in this area. Spaces, for example, is a company that was previously focussed on XR for theme parks and shopping malls, but rapidly developed a solution (in less than 2 months) that provides hybrid virtual experiences to host online meetings that include both conventional screen-based users and HMD users simultaneously. The software is available on a freemium basis as an add on that integrates with platforms like Zoom, Skype and Google Meet and permits users to enter meetings as an avatar. They can also open multiple screens within their VE and view the video streams of participants as individual screens, and present back to an audience making use of a virtual white board. The company also did a deal with ‘selfie avatar’ company Loom.au to permit users to create a personalised avatar with sophisticated animated body language and facial movements that respond to audio cues (SPACES, nd).

Other similar services are in beta stage development, such as MeetinVR (nda), a stand-alone platform that permits fully immersive 3D meetings based on personalised avatars with integrated cocreation tools such as 3D drawing software. The opening lines to the slick corporate promotional video on the home page of their website invite the audience to ‘imagine a new reality where you don’t have to travel to work because you could just teleport there’ (MeetinVR, ndb). The company has also developed a MR version of their application that makes use of high-end Varjo HMDs with see-through capability to allow avatars to merge real and virtual objects into a VE-based meeting space (MeetinVR, ndc; MeetinVR x Varjo XR Collaboration, nd). A number of other companies, such as ENGAGE, rumii, Meeting Room and Glue are framed similarly as meeting platform and offer varying levels of technological sophistication (ENGAGE, nd; Glue Universal Collaboration Platform, nd; meetingRoom, nd; rumii on Oculus Go, nd).

AltspaceVR is a more mature venture that launched its first product in 2015, and was acquired by Microsoft in 2017 in a move likely inspired by Facebook’s aggressive development in XR (Eadicicco, 2017). Their strapline is ‘be there together’, and like MeetinVR, the company offers stand-alone hosting for small-scaled VR events on a freemium model and allow the use of personalised avatars with sophisticated eye tracking technology and compatibility with a range of VR device manufacturers (AltspaceVR, nd). Because the platform is well established, it has been very actively used for meet ups, meetings, shows, demos, product launches and formal education during the COVID-19 crisis. It offers open events like Open Mic Nights, Improv Comedy, Meditation and VR Church and theme-based channels on topics like VR in Education. Facebook Horizon (2019) is the most direct competitor for Altspace VR and is an ongoing development that will be a platform where social media users can meet, play games, explore, socialise and create 3D objects that add to the environment in an expansive virtual environment that Facebook claims will offer advanced immersive interactivity – it is now in the beta phase of maturity. Also worthy of mention is vTime XR, a social network based in Britain that launched in 2015 and since that time has innovated to allow the novel use of AR, VR or 2D interface modes to meet, chat and share media on a free mobile platform (Matney, 2018).

Other more specialised platforms exist, such as training platforms like Sketchbox and Oxford Medical Simulation (OMS), a VR patient management simulation used to train medical professionals (Oxford Medical Simulation, nd; Sketchbox, nd). Collaborative design platforms are also growing in number, such as Softspace, The Wild and SYMMETRY (Softspace, nd; Symmetry Dimensions, nd; The Wild, nd).

It is clear from this brief survey of the larger players, which is by no means comprehensive, that the application of XR technologies to allow for remote social presence is in its early stages but moving towards maturity. Most of these broadly accessible platforms/systems will work with consumer accessible devices such as the Oculus Quest or other major VR headsets, and all set out to address participant need for a stronger feeling of Psi by striving to achieve closer representations of human movement and personalised avatars. In the example of Spaces and MeetinVR, this is explicitly about achieving effective communication through more natural interactions in teleconferences, and typically such companies are framed as future-oriented professional work tools designed to increase efficiencies.

Meanwhile, AltspaceVR and the more ambitious Facebook Horizon are larger social VR projects from major players Microsoft and Facebook. Both form part of these companies’ engagement with a new range of technologies, and particularly in the case of Facebook, a sandbox for the development of new products and services. They are also a safeguard against the emergence of disruptive competitors like vTime XR, and as the very active community of use on AltspaceVR in the time of COVID-19 shows, the versatility and mobile availability of vTime XR might indicate a future model for success in social network platforms. Finally, a larger number of small platforms permit specialised collaborative tools for design and education purposes, and it is likely these applications will grow into more sophisticated remote social presence tools for future use in bespoke work settings.

## Conclusion

During the brief period of the COVID-19 pandemic, companies such as Spaces have raced to meet the need highlighted by a common set of experiences of remote social presence through standard video conferencing. XR has the potential to enhance a sense of presence – of really being there together – in a range of vital human domains, but deeper knowledge and testing are need. In particular, the limits to key categories that define our understanding of XR – immersion and presence – call into question how viable existing presuppositions about this socio-technical context of communication are. Such categorical understandings as Psi rely on a complex array of human experiences, and we appear at present to be scratching the surface of this plausibility despite the advanced nature of such technologies as Facebook’s machine learning–driven Codec Avatar.

We stand at the edge of a period when representations of us in VEs will be achieved with convincing fidelity, and when these new technologies can be designed in a manner that is both cautious of the damage this simulation might do, while embracing their capacity to include and embrace the diversity of human experience. XR technologies are certainly vital to effective remote communication in both personal future of work contexts, and such use cases as strongly immersive video conferencing or shared experience of presentations based on photorealistic or volumetric 3D representations of the participants are among the more obvious and pragmatic of these. But others like persuasive virtual tourism may *need* to become more common in the face of crises such as the lingering presence of pandemic disease or the degrading systems and violent events that accompany a growing climate emergency. Perhaps the most important learning the affordances of these emerging technologies provide is a timely reminder of the fragility of our environment, of the world order and the individuals who rely upon it.
